# Zebrafish, Medaka and Turquoise Killifish for Understanding Human Neurodegenerative/Neurodevelopmental Disorders

**DOI:** 10.3390/ijms23031399

**Published:** 2022-01-26

**Authors:** Kazuki Kodera, Hideaki Matsui

**Affiliations:** 1 Department of Neuroscience of Disease, Brain Research Institute, Niigata University, Niigata 951-8585, Japan; kodera06a031j@gmail.com; 2 Department of Pediatrics, Graduate School of Medical and Dental Sciences, Niigata University, Niigata 951-8510, Japan

**Keywords:** small fish, zebrafish, medaka, turquoise killifish, neurodegenerative disease, Parkinson’s disease, ageing, neurodevelopmental disorder

## Abstract

In recent years, small fishes such as zebrafish and medaka have been widely recognized as model animals. They have high homology in genetics and tissue structure with humans and unique features that mammalian model animals do not have, such as transparency of embryos and larvae, a small body size and ease of experiments, including genetic manipulation. Zebrafish and medaka have been used extensively in the field of neurology, especially to unveil the mechanisms of neurodegenerative diseases such as Parkinson’s and Alzheimer’s disease, and recently, these fishes have also been utilized to understand neurodevelopmental disorders such as autism spectrum disorder. The turquoise killifish has emerged as a new and unique model animal, especially for ageing research due to its unique life cycle, and this fish also seems to be useful for age-related neurological diseases. These small fishes are excellent animal models for the analysis of human neurological disorders and are expected to play increasing roles in this field. Here, we introduce various applications of these model fishes to improve our understanding of human neurological disorders.

## 1. Introduction

When we think of model animals used in medical research, the typical animal is a mouse. Throughout the history of science, many researchers have standardized and advanced experimental procedures using mice, including genetic techniques, biochemical analysis and behavioral analysis. There is no doubt that mice are at the forefront of model animals. In addition to mice, rats, flies and nematodes have a relatively long history as model animals. Among model animals, small fishes such as zebrafish (*Danio rerio*), medaka (*Oryzias latipes*) and turquoise killifish (*Nothobranchius furzeri*) are relative newcomers but are increasingly present (Table 1) [1,2,3,4,5,6,7].

Because fish and mammals, such as humans, diverged so recently in the course of evolution, their anatomical and genetic homologies are remarkably well preserved. In the laboratory, small fishes are excellent model animals with many advantages that mammalian models do not have, such as simple maintenance from embryo to adult, excellent tissue visibility with transparent embryos and larvae, easy handling in laboratory experiments, including molecular biology, biochemistry, histology and so on. This review outlines the characteristics of these small fishes, their relevance to humans, the benefits of using small fishes as model animals for neurological disorders and how small fishes are currently being used in research for neurodegenerative diseases and neurodevelopmental disorders.

## 2. Central Nervous System in Small Fishes

Flies and nematodes, which are used as small model animals, are invertebrates and may be disadvantageous when considered as human disease models. On the other hand, small fishes are vertebrates such as mice and humans, and many of their organ structures are similar to those of humans. The basic structure and function of the central nervous system is conserved from small fishes to humans. In nematodes, for example, the ganglia of the head are sometimes referred to as the “brain”, but they have no direct phylogenetic relationship to the vertebrate brain. Small fishes, on the other hand, have what can accurately be called a brain. Although there are some differences between the brains of small fishes and humans, they are anatomically and functionally similar as a whole. This section describes the anatomical characteristics of the zebrafish brain. An excellent phylogenetic tree of the evolutionary connections among the model animals mentioned above is given in a previous report [6].

The structural and anatomical formation of the central nervous system in zebrafish begins early in development. After the formation of the neural tube at 17 hpf (hours post-fertilization), brain morphogenesis begins. The boundary between the midbrain and hindbrain begins to form, and regions such as the cerebellum and thalamus are developed [8]. Human dopaminergic neurons in the substantia nigra are located in the midbrain, but there are no dopaminergic clusters in the anatomically classified midbrain of zebrafish. There are several clusters of dopaminergic neurons in the neighboring diencephalon, and their formation has begun at 18 hpf [9]. Furthermore, it has been shown that some of the dopaminergic neurons in this diencephalon extend and project long axons to the striatum, and these neurons could be equivalent to human dopaminergic neurons in the substantia nigra [10]. Dopaminergic projections to the forebrain in zebrafish are thought to be homologous to the reward system in mammals [11]. Since the overall composition of dopaminergic neurons is conserved among teleosts, the distribution of dopaminergic neurons in turquoise killifish is similar to zebrafish and medaka. Please refer to the latest report on the analysis of catecholaminergic neurons in the central nervous system of turquoise killifish [12].

The cerebellum has climbing fibers and parallel fibers, and the cell groups present are similar to those of humans, including Purkinje neurons and granule cells [13,14]. Although zebrafish do not have structures corresponding to the deep cerebellar nuclei, cells called eurydendroid cells receive Purkinje cell projections and send efferent projections to various brain regions. Therefore, eurydendroid cells are thought to be functionally homologous to the deep cerebellar nuclei of mammals [15]. In humans, the cerebellum is divided into the vestibulocerebellum, spinocerebellum, and pontocerebellum in terms of phylogeny and functional localization, while the cerebellum of small fishes is mostly regarded as the vestibulocerebellum. We created a functional map of the zebrafish cerebellum and showed that the zebrafish cerebellum contains at least the vestibulocerebellum and spinocerebellum, the details of which can be found in a reference [16]. It is very interesting to determine if there are also cerebellum regions controlling higher brain functions within the telencephalon in small fishes.

Zebrafish also have a telencephalon that corresponds to the human cerebrum, with regions corresponding to the hippocampus and amygdala, which are involved in memory learning and emotional behavior, respectively [17]. In humans, during development, the ventral and dorsal sides of the neural tube form a median constriction, whereas in zebrafish, the dorsal side of the neural tube forms an outward folding [18]. However, unlike humans, zebrafish do not have cortical layer structures. It is important to note that this does not mean that the fish brain does not have structures and functions equivalent to the mammalian cerebral cortex.

The blood–brain barrier (BBB) is also present in zebrafish. Angiogenesis in the hindbrain begins at approximately 20 hpf, and pericytes and glia are found around the vessels by 60 hpf, but the BBB is incomplete until approximately 5–8 dpf (days post-fertilization), allowing various drugs to penetrate the central nervous system [19,20]. The drug can be administered orally or intracorporeally or it can be dissolved in the maintenance water of the larvae and infiltrated into the body tissue by water immersion. This feature is very useful for high-throughput screening using zebrafish larvae. For example, embryos or larvae are individually deposited in each well of 96- and 384-well plates, and various compounds can be dissolved in the water. Thereafter, each fish can be evaluated by gene expression patterns, developmental changes or behavioral analysis. This is less time-consuming and less expensive than the same screening procedure using mammalian models such as mice [21].

One of the major differences between zebrafish and humans is the ability to regenerate the central nervous system, including neurons. When zebrafish are artificially injured in the spinal cord, functional recovery and motor neuron repopulation are observed within 6 to 8 weeks [22,23]. In addition, tissue regeneration of the central nervous system has been observed after artificial damage, even in the telencephalon [24]. The increased regeneration ability of fish, even in the central nervous system, needs to be recognized when using fish as models of human neurological disorders.

Many lines of evidence have been shown using zebrafish. Because zebrafish, medaka and turquoise killifish are closely related teleosts, their major structures in the central nervous system are comparable. A review of these three model fishes including the description of their central nervous system is available [25].

## 3. Ease of Laboratory Management and Experimentation with Zebrafish, Medaka and Turquoise Killifish

### 3.1. Visibility and Light Transmission in Small Fishes

One of the important characteristics of small fishes is their high tissue transparency during embryogenesis and the larval stages. In zebrafish and medaka, the developmental process can be observed outside the body of the parent fish, and the embryos and larvae are transparent, making it easy to observe the developmental process and internal structure. In addition, several mutant lines of zebrafish and medaka are available that are more transparent than the wild type, allowing the internal structure of the fish to see, even in the adult stage [26]. This characteristic allows direct observation of the development and tissue and cell activity, which is compatible with live imaging, and allows us to capture developmental and structural changes in the nervous system in greater detail in vivo. When fluorescent proteins are expressed specifically on the cell of interest, it is possible to observe the development and morphological changes of the neurons of interest over time. Chemical or genetically encoded Ca sensors expressed in neurons allow us to observe the activity of neurons with high temporal and spatial resolution [27]. For example, we showed that cerebellar neural activity during behavior can be observed at the cellular level [16]. A very exciting study has also been reported that expresses Ca sensors in neurons of the whole brain and utilizes the sensor signals as in electroencephalography [28]. Using optogenetics techniques, it is also theoretically possible to activate or suppress the neuronal activity of target neurons at any site [16,29]. A unique method to analyze the pathogenesis of diseases by altering the subcellular localization of target proteins has also been reported [30]. In short, small fishes are unique vertebrates that provide, in a live state, macroscopic observation of the nervous system, microscopic observation of precise neural activity and adaptation of light transmission for optogenetics and other applications.

### 3.2. Ease of Gene Editing with Small Fishes

It is estimated that approximately 71% of human genes have at least one zebrafish gene orthologue [31]. In addition, approximately 84% of the genes known to be associated with human diseases have orthologues in the zebrafish genome [32]. Small fishes have the advantage of simple procedures for gene editing. Efficient genome engineering can be achieved by zinc-finger nucleases (ZFNs) or transcription activator-like effectors (TALENs) in various model organisms, including zebrafish and medaka [33,34,35,36], and the emergence of the CRISPR-Cas9 technique has made it much easier to knockout or edit specific genes in recent years [37,38,39]. In small fishes, oviposition and fertilization occur outside the parents’ body, and gene editing factors are introduced into the fertilized eggs by microinjection, which can then hatch in a petri dish [40]. For example, by injecting GFP mRNA into the embryo, it is possible to observe the results the day after the injection without waiting for days or months [41]. Improved and advanced techniques for gene editing have been reported thus far, and the highly mutagenic CRISPR-Cas9 method enables even injection of F0 embryos mimicking null mutants, making the induction of mutations quick and efficient [42].

Morpholino antisense oligonucleotide-based knockdown of target genes has often been conducted in small fishes, but there can be phenotypic discrepancies between morpholino and knockout phenotypes [43]. This may be due to the presence of morpholino-induced off-target effects or the presence of nonsense-mediated mRNA decay and associated genetic compensation in knockout individuals [44]. In the analysis of gene function in zebrafish, it is strongly recommended to generate mutants and analyze their phenotypes instead of using antisense morpholino oligonucleotides.

The CRISPR-Cas9 system also enables knock-in of the target gene or specific gene variants by means of homology-directed repair (HDR) or other mechanisms [45,46,47,48,49,50]. Stable introduction of exogenous DNA in the zebrafish genome was already achieved in 1988 [51]. Coinjection of the I-SceI meganuclease enzyme together with the transgenic vector carrying the restriction sites [52] or coinjection of tol2 or sleeping beauty transposase with the transgenic vector carrying the transgenic cassette flanked by cis-regulatory repeats [53,54] facilitates germline transmission of the transgene very effectively. These techniques of genome engineering are applicable to zebrafish, medaka and turquoise killifish, but we think it is difficult to conduct microinjection into the eggs of turquoise killifish. This is because of the relatively hard chorion of turquoise killifish compared to zebrafish and medaka, and please kindly see the excellent publications about genome engineering of turquoise killifish [55,56].

If the same thing were to be done with the mouse, gene editing of the embryo would be a complex operation that would require the collection of fertilized eggs from the mated female mouse body, microinjection of the genome-engineering solution into the eggs under clean operations and implantation of the injected eggs into the fallopian tubes of a pseudopregnant mouse, which would be technically much more difficult and expensive. Zebrafish can produce more than 100 eggs per oviposition, making it easy to pick up individuals with the desired gene editing. Zebrafish will mature into adults in 3 months, making it possible to breed the next generation. In the case of medaka and turquoise killifish, the number of eggs per oviposition seems smaller than that of zebrafish, but they lay eggs every day if the condition of the adult fish and aquarium is ideal. Medaka and turquoise killifish can show sexual maturation in approximately 2–3 months and 1 month, respectively. Compared to mice, the cost of subsequent management can also be greatly reduced.

**Figure 1:**
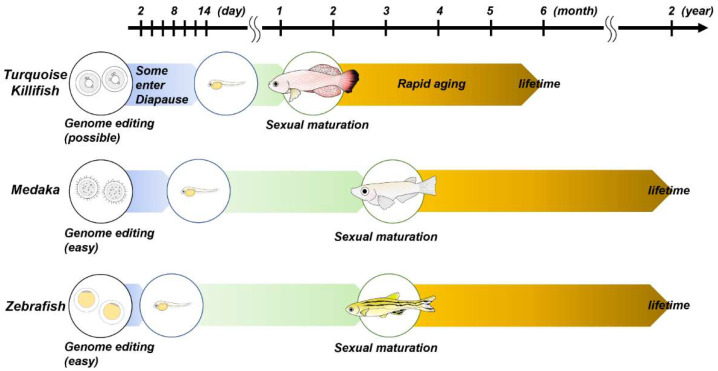
This schema shows the life cycles of zebrafish, medaka and turquoise killifish. For further details, please refer to the main text.

As described above, zebrafish and medaka are the mainstream models for genetic manipulation, while the turquoise killifish provides an excellent model for the studies of aging and age-related disorders (Figure 1). The aging of turquoise killifish is described in the next chapter. When designing a genetic study using small fishes, it is necessary to be conscious of several genetic features. Zebrafish are diploid with 25 chromosomes (25 × 2n) and are close to humans, but their sex chromosomes have not been identified. The mechanism of sex determination is still not fully uncovered, but it has been found that zebrafish sex can be affected by environmental factors [57,58,59]. In contrast, medaka or turquoise killifish sex is determined by XX/XY sex chromosomes [60,61,62]. Vertebrates, including humans, experienced a whole-genome duplication in which the genome doubled twice in our ancestors approximately 500 million years ago. Furthermore, teleosts, including zebrafish, medaka and turquoise killifish, experienced another round of whole-genome duplication [63,64,65]. Therefore, in some genes, there is no 1:1 correspondence between humans and zebrafish, and there can be two orthologous genes. The fact that there are two orthologues means that even if one of the orthologues is knocked out, the other paralogue may be able to compensate for its function. It has also been reported that there are differences in the structure, function and expression patterns between the paralogues of these genes, which might suggest that each gene has its own significance [66]. In some cases, double knockout of those two orthologous genes might be necessary to establish a knockout line with the anticipated phenotypes seen in human diseases or disorders. It is also necessary to check whether the ortholog of the gene of interest exists or not (e.g., the ortholog of *SNCA* is present in medaka but not in zebrafish).

For zebrafish, ZFIN (Zebrafish Information Network, https://zfin.org/; accessed on 30 November 2021) has a database of zebrafish genes, created transgenic lines and mutant lines. Many lines, including ENU mutagenesis products or transgenic reporter lines, are available for purchase and are managed by ZIRC (Zebrafish International Resource Center) or EZRC (European Zebrafish Resource Center). For medaka, please visit the NBRP medaka website (https://shigen.nig.ac.jp/medaka/top/top.jsp; accessed on 30 November 2021) to look for various mutant and transgenic lines. Additionally, NFIN (The *Nothobranchius furzeri* Information Network, https://www.nothobranchius.info/; accessed on 30 November 2021) provides an information on laboratory procedures and a gene database of turquoise killifish.

## 4. Ease of Laboratory Management and Experimentation with Zebrafish, Medaka and Turquoise Killifish

Neurodegeneration is a phenomenon characterized by the progressive loss of neurons, and Alzheimer’s disease, Parkinson’s disease and amyotrophic lateral sclerosis are the major neurodegenerative diseases among humans. They are progressive diseases with a relatively slow chronic clinical course, and the brains show abnormal protein deposition. Each neurodegenerative disease has a relatively selective neuronal loss; for example, dopaminergic neurons and the autonomic nervous system are vulnerable in Parkinson’s disease, and the motor neuron system is selectively lost in amyotrophic lateral sclerosis [67]. Recently, there has been much research on neurodegenerative diseases using the advantageous features of small fishes. To generate and analyze disease models, drug treatment with neurotoxic chemicals, direct microinjection of abnormal proteins, knockout of disease-related genes and knock-in of mutated genes can be used. For Alzheimer’s disease studies, tau transgenic zebrafish [68,69] and zebrafish with direct microinjection of Aβ have been reported [70,71]. For amyotrophic lateral sclerosis studies, TDP-43-, SOD1- or C9orf72-related zebrafish models have been used to analyze the pathogenesis and screen for new drugs [30,72,73,74,75,76]. Here, we discuss Parkinson’s disease; for more information on other neurodegenerative diseases, see other reviews [77,78].

### 4.1. Zebrafish and Medaka Models of Parkinson’s Disease

Parkinson’s disease is a common disease in the elderly and it is characterized by progressive motor impairment, loss of dopaminergic neurons in the substantia nigra and alpha-synuclein-positive inclusion bodies called Lewy bodies. We and others have used a variety of small fishes to study the pathogenesis of Parkinson’s disease. Toxins such as 1-methyl-4-phenyl-1,2,3,6-tetrahydropyridine (MPTP), 6-hydroxydopamine (6-OHDA) and rotenone are known to be toxic to dopaminergic neurons in various animal models. MPTP is a neurotoxin that induces Parkinson’s disease-like symptoms in various animals, including humans. It is metabolized to 1-methyl-4-phenylpyridinium (MPP+) in glial cells and is subsequently incorporated by dopaminergic neurons via dopamine transporters and inhibits the activity of the mitochondrial respiratory chain. Due to this metabolic pathway, dopaminergic neurons are selectively damaged [79,80]. MPTP also induces Parkinson’s disease-like symptoms in medaka. Keeping medaka larvae in MPTP-containing water rapidly induces a reduction in spontaneous swimming movement and a loss of dopaminergic neurons in the diencephalon [81]. MPTP exposure has also been carried out using zebrafish larvae and adults. These studies have demonstrated the effect of MPTP on locomotion and dopaminergic neurons [82,83,84,85,86,87]. 6-OHDA can be taken up by the dopamine transporter, resulting in oxidative stress and selective damage to dopaminergic neurons. 6-OHDA, when administered directly into the cerebrospinal fluid, also induces a loss of dopamine neurons and a decrease in spontaneous swimming movements in medaka [88]. The injection of 6-OHDA into the ventral diencephalon of adult zebrafish or by keeping zebrafish larvae in 6-OHDA-containing water can cause a reduction in dopaminergic neurons and impaired swimming movements [89,90,91,92]. Keeping zebrafish larvae in rotenone-containing water also causes a reduction in dopamine and impaired swimming movements [93,94]. Other chemicals, including proteasome inhibitors [88], ammonium chloride or tunicamycin [95], can induce Parkinson’s disease-like phenotypes in small fishes. These toxin-induced models can be useful for Parkinson’s disease research and drug screening.

Next, we will introduce genetic models of Parkinson’s disease using zebrafish and medaka. It is surprisingly difficult to simulate human Parkinson’s disease by genetic manipulation in mice, which is one of the most representative model animals. For example, even in triple knockout mice with Parkin, PINK1 and DJ-1, the gene products responsible for autosomal recessive familial Parkinson’s disease, there is no dopaminergic neuron loss [96]. Similar to mice, Parkin or Pink1 single knockout medaka does not show dopaminergic cell loss [97,98]. We analyzed Parkin and Pink1 double knockout medaka and found that a loss of dopaminergic neurons occurred, which was not observed in single knockout fish [98]. In the case of zebrafish, single depletion of Pink1 is sufficient to induce the loss of dopaminergic neurons [99,100,101]. DJ-1 knockout zebrafish and medaka models have also been created, which need further pathological evaluations but are promising for the production of new fish models of Parkinson’s disease [102,103,104,105]. 

ATP13A2 is another gene product that is responsible for autosomal recessive early-onset parkinsonism that is characterized by levodopa responsiveness, supranuclear gaze palsy, pyramidal signs and dementia [106]. We created Atp13a2 mutant medaka, which shows a mutation similar to that seen in human patients, and this fish shows loss of dopaminergic neurons with a decrease in cathepsin D activity and fingerprint-like inclusion body formation [107]. Atp13a2 knockout zebrafish also show similar phenotypes [108]. *GBA*, which is also the causative gene of Gaucher disease, is one of the high-risk genes for idiopathic Parkinson’s disease, and Gba knockout medaka and zebrafish exhibit not only dopaminergic neurodegeneration but also alpha-synuclein accumulation [109,110,111]. LRRK2 mutations are a relatively common cause of autosomal dominant familial Parkinson’s disease and they are also related to idiopathic Parkinson’s disease [112,113]. There is a difficulty in understanding such autosomal dominant disease because it might not be clear whether a loss of the normal function of the gene is a major cause of the phenotype or whether a gain of toxic function can explain the disease. There have been several zebrafish models of Lrrk2, but we will await further evaluations and consistent findings [114,115,116,117].

In this way, various models of Parkinson’s disease can be generated by treating zebrafish or medaka with chemicals or by conducting genetic modification of the zebrafish or medaka genome. These models are very useful to analyze the function of molecules related to Parkinson’s disease in vivo and to understand the pathophysiology of human Parkinson’s disease. Furthermore, it has already been widely used in the field of drug discovery. High-throughput screening using the characteristics of zebrafish has picked up many compounds with the potential to improve the pathology of Parkinson’s disease. Please refer to the recent review on drug discovery for major neurodegenerative diseases including Parkinson’s disease [77]. Of course, one limitation of using small fishes as model animals is that there can be a discrepancy between fishes and humans. As is the case with most human diseases, the onset of disease is complicated by a number of factors, such as aging, environmental factors and multifactorial genetic effects. Therefore, we should carefully examine various models including cell lines, small fishes or mammalians and it is also important to investigate human samples. By going back and forth between models such as fish and human samples, we can understand the human pathology more reliably.

### 4.2. Idiopathic Parkinson’s Disease Phenotypes Seen in Turquoise Killifish

Next, we focused on turquoise killifish to understand idiopathic Parkinson’s disease, which is not hereditary and accounts for 90–95% of all Parkinson’s disease cases. Turquoise killifish is a small fish species that lives in ponds, swamps and puddles in Mozambique and other countries [7]. Its habitat has a long dry season and a short rainy season, and during the dry season, the water in which the turquoise killifish lives dries up and the adult fish cannot survive. However, it has been able to survive as a species by adopting a life history in which it spawns drought-resistant eggs in the soil, which hatch during the next or future rainy season. In such a life cycle, a positive selection pressure for anti-ageing does not work [118]. Most likely, turquoise killifish have a short lifespan and exhibit an ageing phenotype in a very short period of time. Specifically, the lifespan of turquoise killifish is approximately four to six months, and around the age of three months, they present various signs of ageing, including organ atrophy, spine curvature and increased levels of senescence-associated beta-galactosidase [119,120,121]. Although Parkinson’s disease is strongly associated with ageing in humans, most experimental animals may not present a sufficient disease phenotype during ageing. We have found that turquoise killifish exhibit degeneration of dopaminergic and noradrenergic neurons and the progression of alpha-synuclein pathology with ageing [122]. These pathological phenotypes are similar to those observed in human Parkinson’s disease. Genetic depletion of alpha-synuclein by CRISPR-Cas9 system ameliorates neurodegeneration, suggesting that alpha-synuclein is not a bystander in the pathogenesis of Parkinson’s disease but is a causative protein of neurodegeneration. The turquoise killifish has the potential to reveal the mechanisms of Parkinson’s disease, especially the majority of cases of idiopathic Parkinson’s disease. This unique fish will also be useful for other age-related disorders in the brain and other organs.

## 5. Human Neurodevelopmental Disorders in Small Fishes

Human neurodevelopmental disorders are diagnosed based on the relative relationship between a person’s behavior and society, such as developmental characteristics and difficulties in social life, not based on genetic diagnosis or biomarkers such as MRI scans [123]. One limitation of using small fishes to study neurodevelopmental disorders is that it is not probable that small fishes will meet the diagnostic criteria for those human neurodevelopmental disorders. Although it is difficult to apply the complex higher-order functions of humans to zebrafish, there have been reports in recent years that zebrafish can be used as a model animal for neurodevelopmental disorders by applying behavioral analysis that imitates human social responses. Furthermore, as has already been mentioned, the utility of small fishes in the laboratory as a model animal for neurodevelopmental disorders has led to many interesting findings in terms of consistent observations from the cellular and molecular scale to tissue, developmental and behavioral analysis.

Autism spectrum disorder (ASD) is one of the most common neurodevelopmental disorders. Although the pathogenesis of ASD has not been established, findings from comprehensive genetic analysis of patients with ASD have been accumulated, and a database of risk genes for the onset of ASD has been created. SFARI (https://gene.sfari.org/; accessed on 30 November 2021), a database operated by the Simon Foundation in the United States, is available for reference. There are currently 1023 registered genes classified by risk intensity. Moreover, genetic factors have been recognized in the pathogenesis of attention-deficit/hyperactivity disorder (ADHD), and in recent years, findings from meta-analyses of genome-wide association analyses have accumulated [124,125,126]. The following is a summary of research reports using zebrafish mutant models of genes thought to be associated with these neurodevelopmental disorders (Table 2).

*DYRK1A* is a serine/threonine kinase that is essential for brain development and function, and overactivation of this protein is observed in Down syndrome [133]. In addition, *DYRK1A* belongs to score 1 in the SFARI database and is considered to be a highly relevant risk gene for ASD. Kim et al. generated and analyzed Dyrk1aa knockout zebrafish, an orthologue of *DYRK1A*. They showed that adult knockout fish showed microcephaly, behavioral analysis showed that anxiety behavior was reduced by the novel tank test, and social interaction was impaired by the shoaling test and social preference test. They concluded that this was an autistic-like behavioral change in fish [127]. In the same way, zebrafish orthologue knockout lines were generated for *SHANK3* and *NRXN2*, which belong to score 1 of ASD risk genes in the SFARI database. SHANK3 is widely expressed in the brain and is mainly involved in the formation of postsynaptic scaffolds and neurotransmission [134]. Liu et al. generated Shank3b knockout zebrafish that showed impaired social interactions by behavioral analysis, and reported reduced expression of Homer1, a SHANK-binding protein, in the adult fish brain [128]. NRXN2 is a transmembrane protein that resides in the presynaptic terminal and is involved in synapse construction and neurotransmitter release mechanisms [135]. NRXN2α knockout mice have been used as a model for autism and have been shown to exhibit increased anxiety-like behavior in assays such as the light/dark box test and the elevated plus maze test [136]. Koh et al. generated Nrxn2a knockout zebrafish and found increased anxiety-like behavior in the novel tank test, suggesting that autism-like behavioral changes also occur in zebrafish [129].

*PER1* is known as a clock gene, and genome-wide association analysis of ADHD patients suggests that this gene is a risk gene for ADHD [124]. Huang et al. created Per1b knockout zebrafish and showed that juveniles were hyperactive, had increased attack frequency in the mirror-image attack test and were rescued by microinjection of *per1b* mRNA. They also showed that the dopamine content was decreased in Per1b knockout zebrafish brains and that the overactive phenotype could be rescued by selegiline (monoamine oxidase B inhibitor) or methylphenidate (dopamine transporter inhibitor, human ADHD treatment). They also analyzed PER1 knockout mice. Similar to the zebrafish model, PER1 knockout mice showed hyperactivity and decreased dopamine content in brain samples, suggesting the possibility that PER1 abnormalities may be involved in dopaminergic neural abnormalities in ADHD [131]. This report is quite impressive because it is suggestive of a highly conserved phenotype among vertebrate species, including behavioral characteristics. 

To summarize how the behavioral characteristics of zebrafish express the symptoms of human neurodevelopmental disorders, “reactivity to anxiety” corresponds to sensory hypersensitivity/sensory deprivation in autism spectrum disorders, “lack of crowding” as a difficulty in social communication and interpersonal interactions and “hyperactivity and aggression” as phenotypes for hyperactivity/impulsivity symptoms in ADHD, can be evaluated in each assay. Even if the anatomical and physiological differences are not clear in a disease model, if some phenotype can be obtained through behavioral analysis, it might be used as a milestone to assess whether some intervention can provide rescue, such as pharmacological high-throughput screening [130,131,132]. What should be carefully considered is its interpretation in behavioral analysis. While the behavioral analysis of mice has a long history and has been standardized by many researchers, the behavioral analysis of zebrafish is still in its development phase. For example, the novel tank test tracks the behavior of zebrafish after they have been transferred to a new tank and aggregates and statistically processes how much time they spent at which water depth and how far they travelled. In this assay, zebrafish first spend time hiding at the bottom of the tank and then they gradually expand their range of activities to the surface. If it is observed that the zebrafish spend less time at the bottom of the tank and immediately start to move closer to the surface, it may have different meanings depending on whether it is explained as “not feeling anxious easily” or “hyperactivity and impulsiveness”. See the references for a list of zebrafish behaviors [137], a summary of behavioral analysis and its limitations and a contrast with behavioral analysis in mice [138,139,140]. Behavioral analysis looks at the habits of fish, but it is necessary to critically consider them when applying them to humans. It would be more convincing if trends in phenotypes could be observed in multiple assays, rather than making assumptions based on the result of a single behavioral analysis. In addition to behavioral analysis, other types of methods that can evaluate stress responses are also being considered; for example, by evaluating the level of cortisol, which is one of the stress hormones [141,142,143]. There are still many unknown aspects of using small fish as a model for human higher brain functions and human neurodevelopmental disorders, and we hope that increasing research will be accumulated.

In addition, zebrafish are also used in the field of psychiatry to analyze schizophrenia and depression. It is very interesting to see the phenotype of zebrafish as a model animal for psychiatric symptoms [144,145]. Even though the fields are different, zebrafish are used in similar ways as described in this review. For more information, refer to other excellent publications [139,146,147].

## 6. Conclusions

In this review, we have discussed the features of zebrafish, medaka and turquoise killifish in the laboratory and the actual analysis of neurodegenerative diseases and neurodevelopmental disorders using these small fishes. In the analysis of human neurological disorders, small fishes are very good model animals and will be further developed in the future. At this point, we need to have a sense of humility towards mammalian model animals. Even if various experimental results are shown in small fishes, if the same thing can be shown in mice, the impact can be greater in mice. To demonstrate the meaning and value of using small fishes, research designs are expected to take advantage of the characteristics of small fishes and their benefits in the laboratory, as described in this review. In addition, we should not forget that we are looking at the human nervous system through small fishes. It may not be clear what the changes in the RNA and proteins in small fishes in the context of human diseases and disorders mean, if we only pay attention to small fishes. The same thing is applicable to the meaning of the changes in morphology and physiological functions at the organ level and the meaning of changes in behaviors obtained through behavioral analysis. The meaning of the results obtained from small fishes will become clear when the results are bridged to mammalian model animals such as mice and then to human analysis. If such a relationship can be established between small fishes and other samples, these fishes can become increasingly powerful and useful tools for solving human neurological disorders.

## Figures and Tables

**Table 1:** Characteristics of zebrafish, medaka and turquoise killifish.

	Zebrafish	Medaka	Turquoise Killifish
	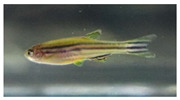	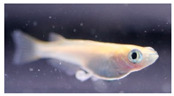	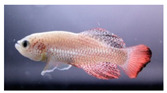
Scientific name	*Danio rerio*	*Oryzias latipes*	*Nothobranchius furzeri*
Lifespan	2–5 years	2–5 years	4–6 months
Generation time	2–3 months	2–3 months	1 month
Water temperature	27–29 °C (20–33 °C)	25–28 °C (0–40 °C)	28 °C (20–35 °C)
Genome size	1700 Mbp	800 Mbp	1500 Mbp
Number of chromosomes	25 chromosomes (2n = 50)	23 (West Korea, China) or 24 (Japan, East Kore) chromosomes (2n = 46 or 48)	19 chromosomes (2n = 38)
Time to hatch	2 days	7–8 days	12–14 days (Longer if embryos enter diapause, from several months to several years)
Sex determination	Environment?	XY/XX	XY/XX
Difficulty of breeding	simple	simple	difficult

The characteristics of zebrafish, medaka and turquoise killifish are described. For further details, please refer to the main text.

**Table 2:** Studies using behavioral analysis in zebrafish with gene mutations associated with neurodevelopmental disorders.

Fish	Disorder in Human	Behavioural Assay	Phenotype	Ref.
Dyrk1aa KO zebrafish	ASD	Novel tank test	↓ anxiety-like behaviour (↓ spending time in the bottom ↓ freezing time)	[127]
		Shoaling test	impaired social preference (↑ independently from one another)	
		Social preference test	impaired social preference (↓ spending time near social zone)	
Shank3b KO zebrafish	ASD	Locomotor activity screening	↓ spontaneous activity ↓ responses to changes in illumination	[128]
		Shoaling test	impaired social preference (↑ swimming away from group, ↑ spending outside group)	
		Social preference test	impaired social preference (↓ frequency of social contact)	
Nrxn2a KO zebrafish	ASD	Novel tank test	↑ anxiety-like behaviour (↑ spending time in the bottom, ↑ freezing and thigmotaxis)	[129]
Cntnap2ab KO zebrafish	ASD	Locomotor activity screening	↑ nighttime activity	[130]
Per1b KO zebrafish	ADHD	Locomotor activity screening	↑ locomotor activities ↑ swimming velocity	[131]
		Image-attack assay	↑ attack mirror image (nearly continuously, failing to break off)	
Chmp7 KO zebrafish	ADHD	Locomotor activity screening	↑ average time spent moving each hour	[132]

Various mutant zebrafish lines that target risk genes of neurodevelopmental disorders have been reported, and among them, we have highlighted studies conducting behavioral analysis. Please refer to the main text for details. KO: knockout. ASD: Autism spectrum disorder. ADHD: Attention-deficit/hyperactivity disorder.

## Data Availability

Data and tools described in this manuscript are available upon request.
